# Colloid carcinoma derived from intraductal papillary mucinous neoplasm of the pancreatic head with calcification: A case report and literature review

**DOI:** 10.1186/s40792-021-01286-5

**Published:** 2021-09-06

**Authors:** Ryoichi Miyamoto, Katsumi Amikura, Shinichi Matsudaira, Hiroyuki Ishida, Toshiro Ogura, Amane Takahashi, Atsushi Kihara, Hiroaki Kanda, Yoshiyuki Kawashima

**Affiliations:** 1 Department of Gastroenterological Surgery, Saitama Cancer Center, 780 Komuro, Ina-machi, Kita-Adachi-gun, Saitama, 362-0806 Japan; 2 Department of Pathology, Jichi Medical University, Tochigi, Japan; 3 Department of Pathology, Saitama Cancer Center, Kita-Adachi-gun, Saitama, Japan

**Keywords:** IPMN, Calcification, Osseous metaplasia, Colloid carcinoma, Chronic pancreatitis

## Abstract

**Background:**

Colloid carcinoma derived from intraductal papillary mucinous neoplasm (IPMN) of the pancreatic head with prominent calcification is exceedingly rare. Only a few studies about this entity have been reported in the literature. Therefore, its biological behavior, appropriate treatment modalities, and overall patient prognosis remain largely unclear. In this report, we present a case of a resected colloid carcinoma derived from IPMN with prominent calcification. In addition, we review the relevant literature and discuss the clinical management of colloid carcinoma derived from IPMN with prominent calcification, including the histopathological features.

**Case presentation:**

A 75-year-old man presented with a pancreatic tumor measuring 58 mm on the head of the pancreas that was incidentally detected by abdominal ultrasonography. Abdominal computed tomography and endosonography revealed a multilobular cystic lesion with a 17 mm mural nodule in the pancreatic head. Furthermore, prominent calcification was observed on part of the cyst wall. Magnetic resonance cholangiopancreatography showed a multilobular cyst in the branch duct lacking communication between the cystic lesion and the main pancreatic duct. Thus, the lesion was diagnosed as intraductal papillary mucinous carcinoma (IPMC) with a preoperative classification of T1N0M0 stage IA according to the 8th Union for International Cancer Control (UICC) guidelines, and the patient underwent conventional pancreatoduodenectomy. The resected specimen was microscopically found to contain colloid carcinoma, probably derived from IPMN. In addition, marked calcification was confirmed in the partition wall of the cystic mass. The postoperative course was uneventful, and no evidence of recurrence or metastasis was observed after 10 months of follow-up.

**Conclusions:**

We consider that colloid carcinoma derived from IPMN should be differentially diagnosed as a pancreatic multilobular cystic lesion with prominent calcification that shows no sign of systemic chronic pancreatitis.

## Introduction

Intraductal papillary mucinous neoplasms (IPMNs) typically present with cystic dilation of the pancreatic duct-associated mucin production and variable cellular atypia [1]. Pancreatic calcification is generally considered to be a pathognomonic sign of chronic pancreatitis. Therefore, pancreatic calcification with IPMN is an exceedingly rare clinical entity that shows no sign of systemic chronic pancreatitis [2, 3]. Only a few reports on calcification associated with IPMN have been reported in the literature [4, 5]. Therefore, its biological behavior, appropriate treatment modalities, and overall patient prognosis remain largely unclear.

In this report, we present a case of a resected colloid carcinoma derived from IPMN with prominent calcification. In addition, we review the relevant literature and discuss the clinical, radiological, and histological findings of IPMN associated with calcification to gain insights into the pathogenesis of calcification in IPMN.

## Case presentation

A 75-year-old man presented with a pancreatic tumor measuring 58 mm on the head of the pancreas that was incidentally detected by abdominal ultrasonography. The patient had no significant past medical history, and his general physical examination was normal. No abnormal laboratory findings were observed.

**Fig. 1:**
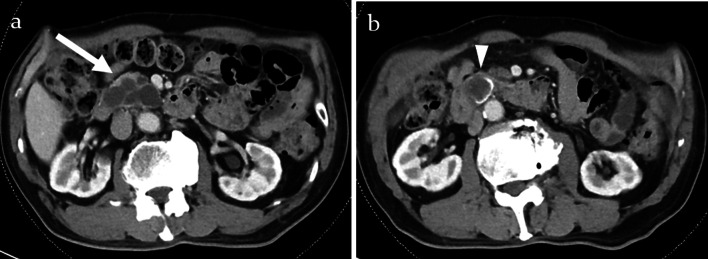
**a** Enhanced abdominal computed tomography (CT) revealed a 58 mm multilobular cystic lesion with a 17 mm mural nodule (white arrow) in the pancreatic head. **b** Prominent calcification was observed on part of the cyst wall (white arrowhead)

Enhanced abdominal computed tomography (CT) revealed a 58 mm multilobular cystic lesion with a 17 mm mural nodule in the pancreatic head. Furthermore, prominent calcification was observed on part of the cyst wall (Fig. 1).

**Fig. 2:**
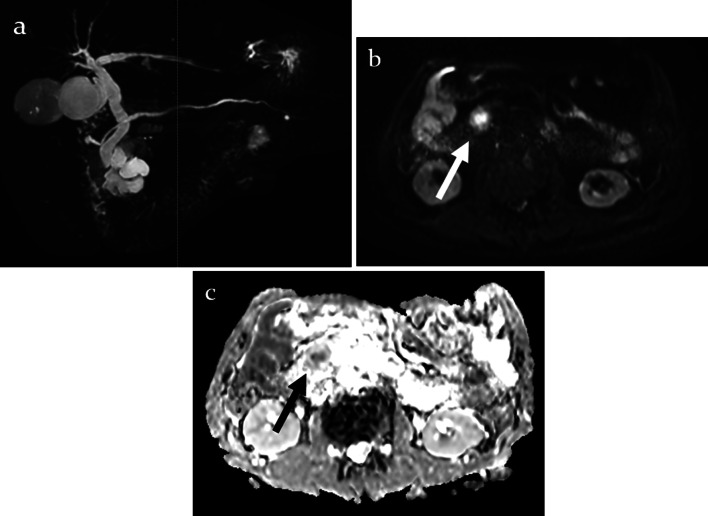
**a** Magnetic resonance cholangiopancreatography (MRCP) showed a multilobular cyst in the branch duct with communication between the cystic lesion and the main pancreatic duct. **b** Diffusion-weighted imaging (DWI) showed an area of hyperintensity in the mural nodule (white arrow). **c** Apparent diffusion coefficient (ADC) was decreased (black arrow)

Magnetic resonance cholangiopancreatography (MRCP) showed a multilobular cyst in the branch duct with communication between the cystic lesion and the main pancreatic duct. Diffusion-weighted imaging (DWI) showed an area of hyperintensity in the mural nodule in the pancreatic head, but the apparent diffusion coefficient (ADC) was decreased. In terms of the distal pancreas other than calcification of the pancreatic head cyst wall, speckle images were not observed on DWI or ADC (Fig. 2).

**Fig. 3:**
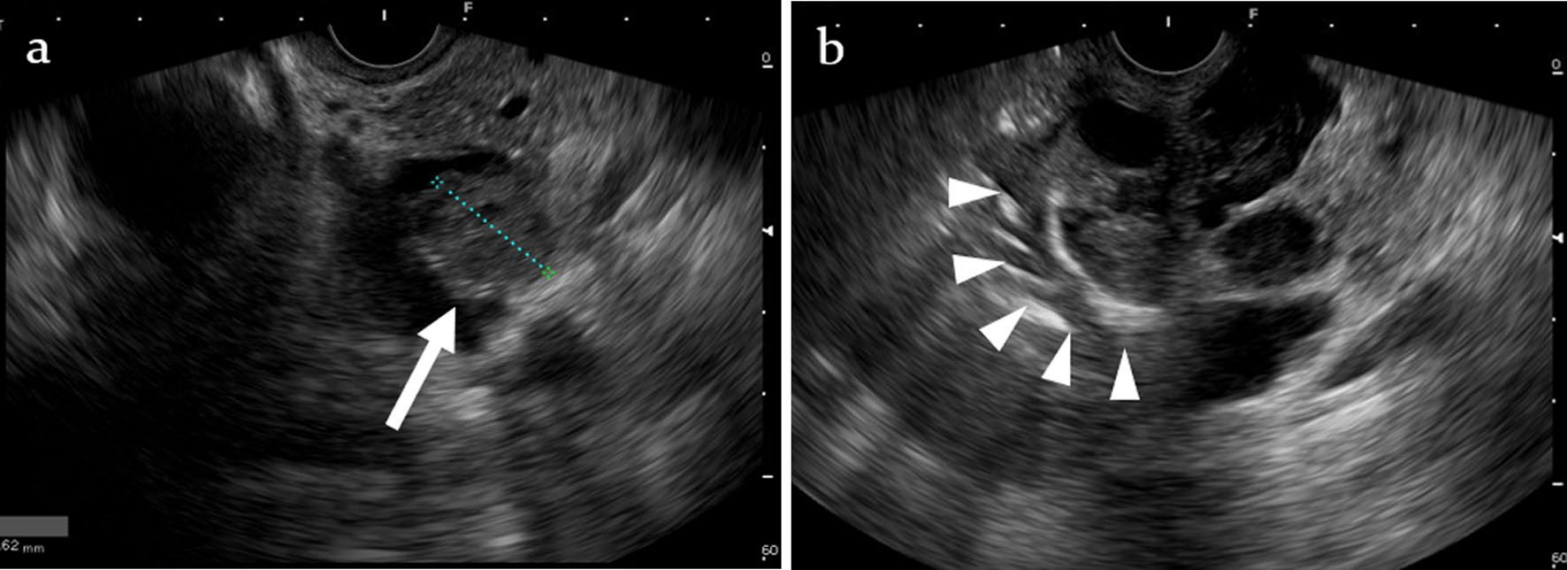
**a** Endoscopic ultrasound (EUS) revealed a multilobular cystic lesion with a 17 mm mural nodule (white arrow) in the pancreatic head. The blood flow signal in the mural nodule was not observed. **b** Prominent calcification with acoustic shadow was observed on part of the cyst wall (white arrowheads)

Endoscopic ultrasound (EUS) revealed a multilobular cystic lesion with a 17 mm mural nodule (white arrow) in the pancreatic head. Furthermore, prominent calcification was observed on part of the cyst wall. In contrast, calcification suggestive of chronic pancreatitis other than calcification of the pancreatic head cyst wall was not observed in the distal pancreas. EUS also revealed communication between the cystic lesion and the main pancreatic duct (Fig. 3).

Thus, the lesion was preoperatively diagnosed as IPMC of the pancreatic head and classified as T1N0M0 stage IA according to the 8th Union for International Cancer Control (UICC) guidelines.

Subsequently, the patient underwent pancreaticoduodenectomy and regional lymph node dissection. At the postoperative follow-up, the patient was followed with adjuvant chemotherapy adjuvant chemotherapy using S-1, which is an oral fluoropyrimidine derivative that combines tegafur with 2 modulators of 5-fluorouracil metabolism, 5-chloro-2,4-dihydroxypyridine and potassium oxonate. The patient exhibited no evidence of recurrence or metastasis after 10 months of follow-up.

**Fig. 4:**
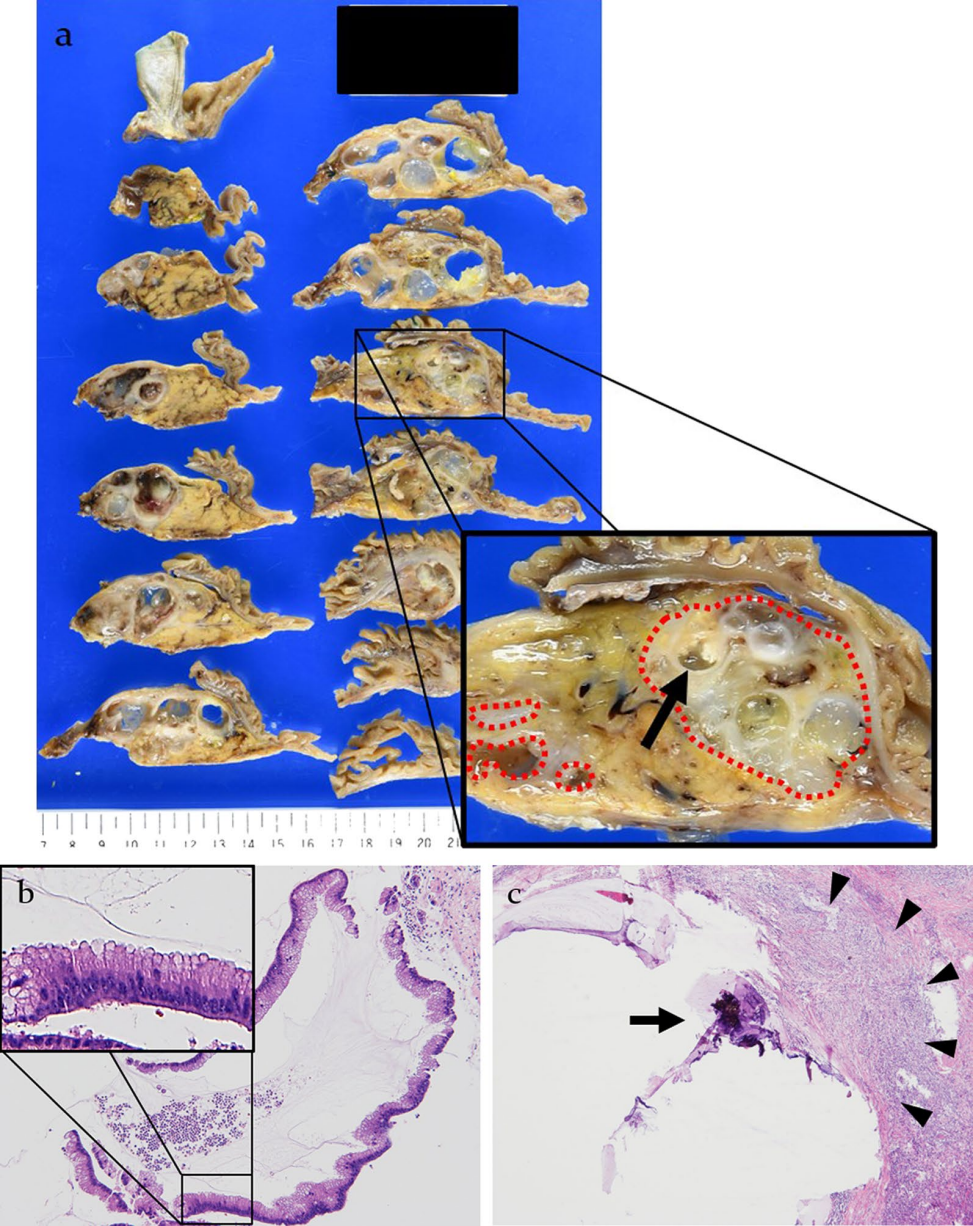
**a** The 60 × 50 mm multilobular cystic lesion was observed in the pancreatic head. The cut surface of the resected specimen in the pancreas head showed a large multilobular cystic mass filled with mucin and mural nodules. The partition wall of the mass was exceedingly hard, demonstrating calcification (black arrow). The precise area of the carcinoma component was described using the red dotted line annotation method. **b** Tumor cells were composed of tall papillae lined by columnar cells with pseudostratified nuclei and basophilic cytoplasm with variable amounts of apical mucin. Hematoxylin and eosin staining, ×50. **c** Calcification was observed on a part of the wall forming the mucin pools (black arrow). Infiltration of inflammatory cells into part of the wall forming the mucin pools was also observed (black arrowheads). Hematoxylin and eosin staining, ×50

Histologically, a 60 × 50 mm multilobular cystic lesion was observed in the pancreatic head. The cut surface of the resected specimen from the pancreas head showed a large multilobular cystic mass filled with mucin and mural nodules. The partition wall of the multilobular cystic lesion was exceedingly hard, demonstrating calcification (Fig. 4).

Microscopically, the tumor displayed the presence of large pools of extracellular mucin containing scattered neoplastic cells. The tumor cells were composed of tall papillae lined by columnar cells with pseudostratified nuclei and basophilic cytoplasm with variable amounts of apical mucin. Calcification was observed on part of the wall forming the mucin pools. Infiltration of inflammatory cells into part of the wall forming the mucin pools was also observed (Fig. 4). In terms of metastasis in the resected lymph nodes, lymph node involvement was not observed.

**Fig. 5:**
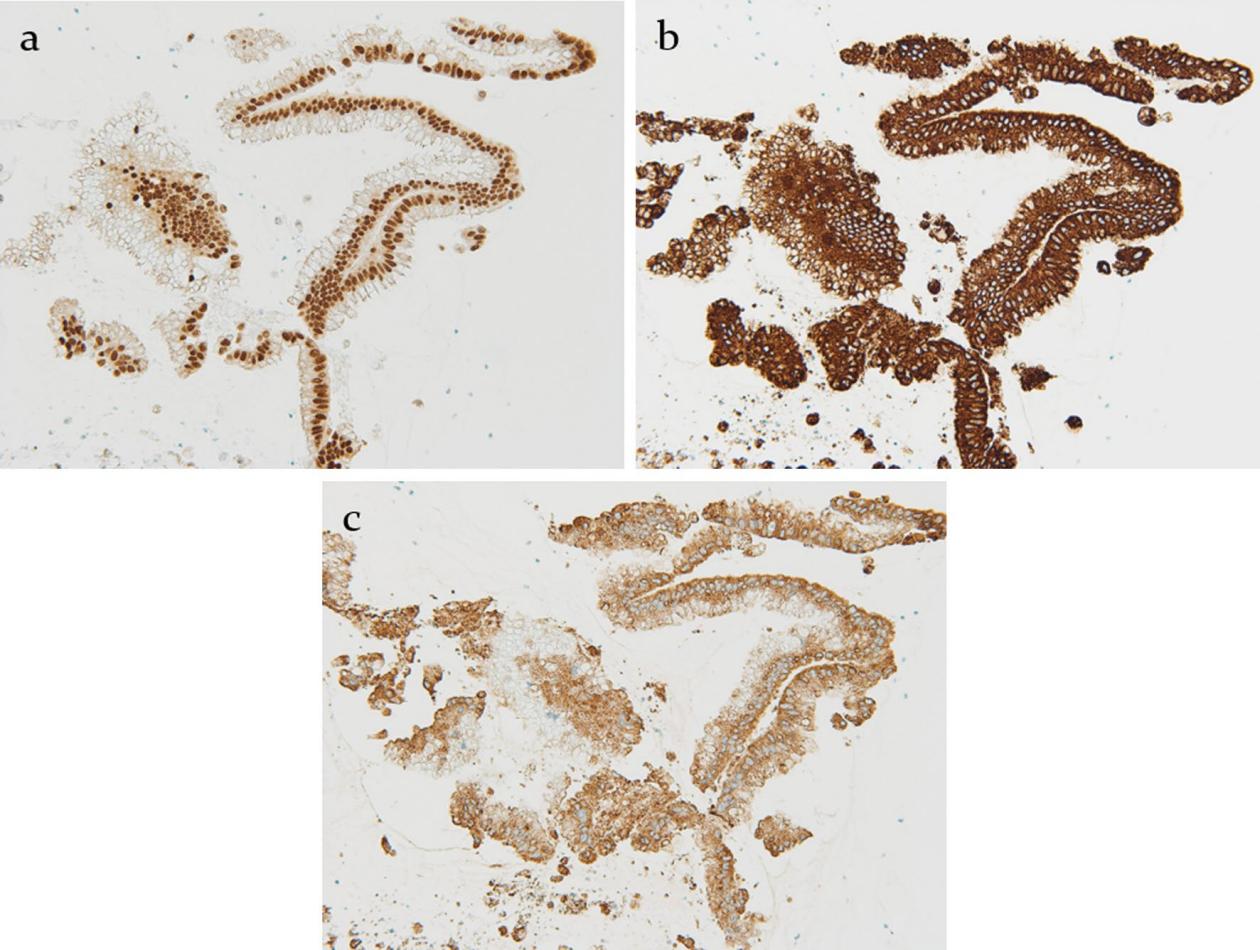
**a**–**c** Cancer cells exhibited strong expression of “intestinal” differentiation markers, including CDX2, ×50 (**a**), MUC2, ×50 (**b**) and MUC5AC, ×50 (**c**)

Immunohistochemically, the cancer cells exhibited strong expression of “intestinal” differentiation markers, including CDX2, MUC2, and MUC5AC (Fig. 5).

Therefore, the final histopathological analysis of the specimen revealed colloid carcinoma derived from the intestinal type of IPMN was T1N0M0 stage IA according to the 8th UICC guidelines.

## Discussion

We present a rare case of a patient with surgically resected colloid carcinoma derived from IPMN of the pancreatic head with prominent calcification.

Colloid carcinoma represents approximately 1% of pancreatic cancers [6, 7]. They are described as a histopathological variant of ductal adenocarcinoma, which is characterized by the presence of large pools of extracellular mucin containing neoplastic cells. The mucin component comprises at least 50% colloid carcinoma (according to the definition by the World Health Organization) or at least 80% of the tumor (according to the US Armed Forces Institute of Pathology) [6, 7]. In almost all cases, colloid carcinoma develops from pre-existing intraductal papillary mucinous neoplasms, especially those forming intestinal-type papillae, and is characterized by MUC2 expression, which is rarely found in pancreatic ductal carcinoma [6, 7]. In the present case, the tumor exhibited that the mucin component comprises at least 50% of colloid carcinomas. Furthermore, the cancer cells exhibited strong expression of “intestinal” differentiation markers, including CDX2, MUC2, and MUC5AC.

IPMNs typically present with cystic dilatation of the pancreatic duct associated with mucin production and variable cellular atypia. Although extensive pancreatic calcification is generally considered to be a pathognomonic sign of chronic pancreatitis, it may also occur simultaneously with IPMN, leading to diagnostic confusion [1, 2, 8].

In terms of the relationship between IPMN and chronic pancreatitis, previous studies reported that twelve to sixty percent of patients with IPMN have a history of symptoms that lead to a diagnosis of chronic pancreatitis, and approximately 2% of all diagnoses of chronic pancreatitis are associated with IPMN [9, 10]. However, few reports of patients with concomitant calcifying pancreatitis and IPMN exist [2, 8]. Talamini G et al. reported that in a cohort study of 473 patients suffering from chronic pancreatitis, only 6 were found to have IPMN during follow-up, but none had calcifications [10]. According to our review of 16 previous case reports, the mean age of patients with IPMN with calcification was 68 years (53–75 years), and 50% were male. The mean tumor size in patients with focal disease was 33.5 mm (1.0–60.0 mm). Fifteen of 16 patients with IPMN with calcification had no history of pancreatitis before presentation with IPMN. Thirteen of 16 patients underwent surgical resection. Histologically, almost all patients had intraluminal calcification in the main pancreatic duct and side branches, often within inspissated mucus, indistinguishable from that in chronic calcifying pancreatitis [2, 8].

Questions regarding the mechanisms by which prominent calcification develops on part of the multilobular cystic wall in IPMN remain unanswered. There are 3 possible explanations for the calcification seen in IPMN.

First, chronic partial ductal obstruction by mucin plugs predisposes patients to intraductal calcification. Previous reports proposed that IPMN may be responsible for pancreatic calcification due to chronic partial ductal obstruction by mucin plugs [2, 8]. Second, the consequences of tumoral calcification include long-term mucus retention. Sanerkin thought that mucinous stromal infiltration was associated with osseous metaplasia and, similar to dystrophic calcification, frequently occurred close to tumor necrosis [11]. Third, local inflammatory signals in the cancer microenvironment mediate the differentiation of endothelial-derived mesenchymal stem cells into chondrocytes and osteoblasts to induce calcification. Medici et al. reported that local inflammatory signals and/or other changes in the tissue microenvironment mediate the differentiation of endothelial-derived mesenchymal stem cells into chondrocytes and osteoblasts to induce heterotopic ossification [12].

In the present case, large pools of extracellular mucin and local infiltration of inflammatory cells in the cystic wall were observed. Therefore, we assumed that the consequences of tumoral calcification, including long-term mucus retention and/or local infiltration of inflammatory cells, contribute to calcification of the multilobular cystic wall in IPMN. In addition, the results of immunostaining, such as anti-osteocalcin antibody, to confirm the presence of chondrocytes and osteoblasts may also be considered to pursue its potential etiology with calcification in the IPMN cyst wall.

In terms of the clinical management of patients with calcification in IPMN, evidence-based clinical management or treatment strategies remain poorly understood. However, surgical resection should be recommended for conventional high-risk IPMNs. Colloid carcinoma demonstrated an improved 5-year overall survival rate of 40–60% compared with tubular carcinomas, which behave similarly to pancreatic ductal adenocarcinoma with a 5-year overall survival rate of 10–20% [13, 14]. A previous case report showed that patients with calcification in IPMN who underwent surgical resection had a favorable postoperative prognosis over the 40-month follow-up period [5]. Similarly, in the present case, the patient underwent pancreaticoduodenectomy and regional lymph node dissection. No evidence of recurrence or metastasis was observed after 10 months of follow-up. Therefore, if the preoperative diagnosis or patient’s condition allows, surgical resection should be considered as one of the treatment options for calcification in IPMN.

## Conclusions

We presented a rare case of a patient with surgically resected colloid carcinoma derived from IPMN of the pancreatic head with prominent calcification. Because of the rarity of colloid carcinoma derived from IPMN with prominent calcification, the prognosis and details of its biological behavior remain unclear. We suggest that colloid carcinoma derived from IPMN should be considered one of the differential diagnoses of pancreatic multilobular cystic lesions with prominent calcification that shows no sign of systemic chronic pancreatitis.

## Data Availability

The data that support the findings of this study are available from the corresponding author.
